# Imbalance of tissue inhibitors of metalloproteinases (TIMP) – 1 and – 4 serum levels, in patients with inflammatory bowel disease

**DOI:** 10.1186/1471-230X-8-55

**Published:** 2008-11-26

**Authors:** Andreas N Kapsoritakis, Anastasia I Kapsoritaki, Ioanna P Davidi, Vasilios D Lotis, Anastasios C Manolakis, Petros I Mylonis, Aikaterini T Theodoridou, Anastasios E Germenis, Spyros P Potamianos

**Affiliations:** 1 Department of Gastroenterology, University of Thessaly, School of Medicine, Larissa, Greece; 2 Department of Immunology and Histocompatibility, University of Thessaly, School of Medicine, Larissa, Greece

## Abstract

**Background:**

Tissue inhibitors of metalloproteinases (TIMPs) play a key role in tissue degradation and remodeling. Since chronic inflammation is associated with tissue remodeling in inflammatory bowel disease (IBD), we evaluated serum TIMP-1 and TIMP-4 levels in IBD patients, in comparison with healthy controls (HC).

**Methods:**

TIMP-1, TIMP-2 and TIMP-4 serum levels were determined in 53 patients with ulcerative colitis (UC), 52 patients with Crohn's disease (CD) and 50 HC, by means of commercially available enzyme-linked immunosorbent assays. The levels of TIMPs were evaluated with regard to the levels of inflammatory markers, such as C reactive protein (CRP) and serum amyloid A (SAA) and the clinical characteristics of patients, so that potential correlations could be recorded.

**Results:**

Mean serum TIMP-1 levels were 414.9 ± 17.6 ng/mL in UC patients, 446.1 ± 22.8 ng/mL in CD patients and 296.5 ± 20.6 ng/mL in HC. UC and CD patients had significantly higher serum TIMP-1 levels when compared to HC, (p < 0.0001 in both groups). Mean serum TIMP-1 levels were significantly higher in patients with active IBD (450.5 ng/mL) in comparison with patients with inactive disease (417.3 ng/mL, p = 0.03). Moreover, males showed significantly higher mean serum TIMP-1 levels (399.8 ng/mL), compared to females (368.5 ng/mL, p = 0.04). Mean serum TIMP-2 levels did not differ between UC and CD patients or HC (p > 0.05 in all cases). Mean serum TIMP-4 levels were 1761.2 ± 67.7 pg/mL in UC patients, 1708.1 ± 73.4 pg/mL in CD patients and 5573.4 ± 1246.3 pg/mL in HC. UC and CD patients had significantly lower serum TIMP-4 levels when compared to HC (p = 0.008 and p = 0.02 respectively). Mean serum TIMP-4 levels were significantly lower in males (2772.9 pg/mL), compared to females (3299.0 pg/mL, p = 0.01). In addition, CRP levels showed a statistically significant correlation with TIMP-1 (r = 0.247, p = 0.01), and TIMP-4 levels (r = 0.217, p = 0.03). Similarly, there was a statistically significant correlation between SAA levels and both TIMP-1 (r = 0.264, p = 0.008) and TIMP-4 serum levels (r = 0.212, p = 0.03).

**Conclusion:**

An imbalance between TIMP-1 and TIMP-4 serum levels is present in IBD patients. TIMP-1 levels could be used not only for diagnostic purposes but also for the assessment of activity in IBD. Gender tends to influence TIMP-1 and TIMP-4 serum levels. These new findings bring into question the potential role of TIMPs in IBD, thus underlining the need for future studies which could offer new insight into this matter.

## Background

Inflammatory bowel diseases (IBD), are characterized by an inflammatory cascade of mediators capable of degrading and modifying bowel wall structure as well as inducing the formation of chronic inflammatory lesions of the digestive tract. The inflammatory cell infiltrate observed in chronic mucosal inflammation is associated with changes in epithelial proliferation and migration and accompanied by intensive remodeling of the subepithelial connective tissue, which in turn leads to increased turnover of extracellular matrix (ECM) components [1,2]. A disturbance in the balance between synthesis and degradation of ECM components can result either in progressive organ destruction, as seen in ulcer formation, or fibrosis due to excessive deposition of collagen [1,2].

Metalloproteinases (MMPs) and tissue inhibitors of metalloproteinases (TIMPs) show a regulated and coordinated pattern of activity which allows tissue degradation and remodeling but at the same time it prevents tissue damage [3,4]. TIMPs are the natural inhibitors of MMPs found in most tissues and body fluids. Currently, four TIMPs (TIMP-1, -2, -3 and -4) are identified. Like MMPs, the expression of TIMPs in the tissue is also controlled to maintain a balance in the metabolism of the ECM [4]. Disruption of this balance may result in a number of pathogenic processes. The 21–34 kDa proteinic molecules of TIMPs express an inhibitory activity which is facilitated by their ability to form high-affinity non-covalent complexes with the carboxyl-terminal domains of pro-MMPs [3]. TIMP-1, TIMP-2 and TIMP-4 are present in soluble forms, while TIMP-3 is tightly bound to the matrix [4]. Numerous studies have indicated that, independently of MMP inhibition, TIMPs are multifunctional proteins involved not only in tissue remodeling and wound healing but also in many other physiological and pathological processes such as angiogenesis, steroidogenesis, hematopoiesis, cell growth and cell survival [4].

The TIMP-1 levels in the mucosa and plasma of IBD patients have been shown to be elevated, in previous studies [5-8]. Moreover, the TIMP-1 plasma levels demonstrate a significant positive correlation with the endoscopic degree of mucosa injury, disease activity and C-reactive protein concentration, in UC patients [8]. In contrast, TIMP-2-mRNA levels remained unchanged and no remarkable expression of TIMP-2 was observed in inflamed mucosa of IBD patients [6,7]. To our knowledge, no data regarding TIMP-4 in IBD have been previously presented. On the other hand, the assessment of serum TIMPs levels has not been performed to date in IBD patients. The aim of the present study was to evaluate serum levels of TIMPs -1, -2 and -4 in IBD patients and to examine possible correlations with known inflammatory markers as well as with the clinical characteristics of patients.

## Methods

### Patients (Table 1)

**Table 1:** Clinical characteristic of the patients with inflammatory bowel disease

	UC	CD
Patients no	53	52
Mean age ± SD (range), years	49.8 ± 13.9 (20–79)	36.6 ± 13.6 (17–69)
Mean duration of disease ± SD (range), months	85.9 ± 81.8 (1–348)	72.4 ± 68.6 (1–388)
Male	30	21
Female	23	31
Current smokers	5	32
Ex smokers	20	2
Never smokers	28	18
Disease extent (UC)		
Proctitis	10	
Left-sided colitis	15	
Extensive colitis	28	
Disease location (CD)		
Ileum		17
Colon		4
Ileum + colon		31
Disease behavior (CD)		
Inflammatory		32
Strictrurizing		8
Penetrating		12
Active disease	22	17
Inactive disease	31	35
Extraintestinal manifestations	24	29
Current treatment*		
5-ASA	51	51
Steroids	28	43
Enemas (5-ASA and/or steroids)	27	5
Immunosuppressive agents	13	21
Metronidazole	11	28
Infliximab	4	15
Parenteral nutrition	2	1

*Some patients received > 1 drug.

One hundred and five consecutive IBD patients followed up at the Department of Gastroenterology of the University Hospital of Larissa, in Thessaly, participated in this study. Details on the clinical characteristics of the patients included in the study are shown in table 1. All patients had a definitive diagnosis UC or CD confirmed by clinical, endoscopic, radiological and histological work-up. Disease activity in CD patients was determined by the Crohn's Disease Activity Index (CDAI) [9]. The cut-off point for active disease was a score greater than 150. Disease activity in patients with UC was assessed by the Clinical Colitis Activity Index (CCAI) [10]. A score greater than 4 on a scale of 0–16 was required for the disease to be considered active. Evaluation of disease activity was performed at the time of serum collection. These patients were compared with the control group, which consisted of 50 healthy blood donors (healthy controls, HC), 33 of them were men, with a mean age (± SD) of 42.6 (± 9.8) years. Apart from routine laboratory parameters, serum amyloid A (SAA) and C reactive protein (CRP) were determined in all patients. Informed consent was obtained from all patients. Patients with myeloproliferative disorders, malignancies, or autoimmune diseases were excluded from the study.

### Laboratory tests

Blood samples were collected in serum separator tubes and were centrifuged for 15 min at 1000 × g, right after a 30-minute clotting period had elapsed. All serum samples were stored at -25°C until assayed. As far as the quantitative serum determination of TIMP-1, TIMP-2 and TIMP-4 is concerned, commercially available solid phase enzyme-linked immunosorbent assay (ELISA) kits (R&D Systems, Abingdon, UK) were used. The minimum detectable doses of TIMP-1, TIMP-2 and TIMP-4 were 0.08 ng/mL, 0.011 ng/mL and 4.91 pg/mL respectively. The intra- and inter-assay coefficient variations for TIMP-1, TIMP-2 and TIMP-4 were below 5%, 4.4% and 5.6% and below 4.9%, 7.3% and 9.2% respectively (n = 20 and n = 40 respectively). All samples were assayed in duplicate.

Serum CRP and SAA measurements were performed by immunonephelometry with the Behring Nephelometer Analyzer II (BNII), using the N High Sensitivity kit and the N Latex SAA kit (Dade Behring Gmbh, Germany), respectively. The appropriate control and standard sera were provided by the same company, and used according to the manufacturer's instructions.

### Statistical analysis

All results are expressed as mean ± SEM. Comparisons between two groups were made by the Mann-Whitney U test. A linear regression or a Spearman r-test was used to assess correlation. All analyses were two-tailed and conducted using the computer-based statistic software program SPSS 15.0 for Windows. A level of p < 0.05 was considered statistically significant.

## Results

### TIMPs serum levels (Figure 1; Figure 2; Figure 3)

**Figure 1:**
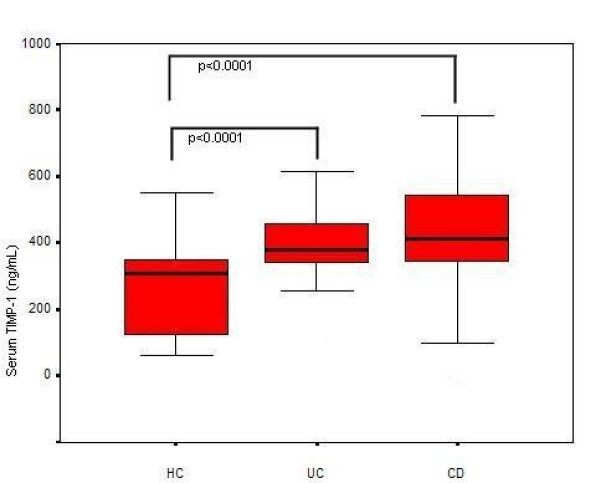
Bar graph showing TIMP-1 levels in UC, CD patients and HC and the statistical significance of mean differences.

**Figure 2:**
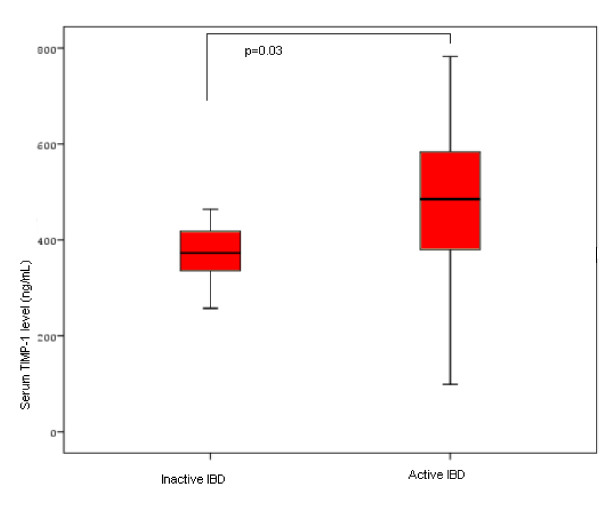
Bar graph showing TIMP-1 levels in patients with active and inactive IBD.

**Figure 3:**
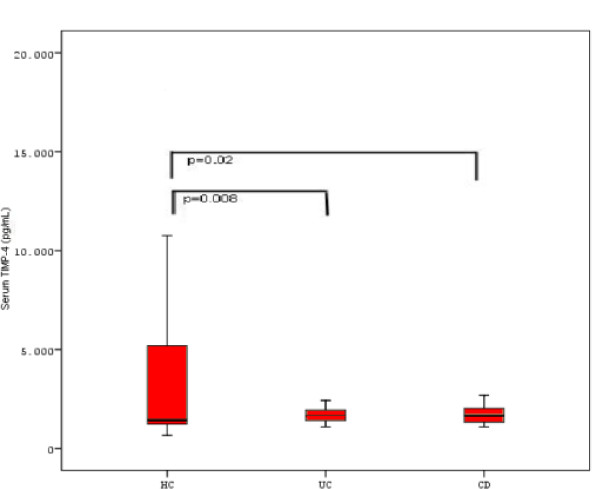
Bar graph showing TIMP-4 levels in UC, CD patients and HC and the statistical significance of mean differences.

Mean ± SEM serum TIMP-1 levels were 414.9 ± 17.6 ng/mL (median: 385.8, range: 70.3–749.9) in UC patients, 446.1 ± 22.8 ng/mL (median: 414.4, range: 9.0–782.1) in CD patients and 296.5 ± 20.6 ng/mL (median: 308.8, range: 63.1–552.0) in HC, (Figure 1). UC and CD patients had significantly higher serum TIMP-1 levels compared to HC (p < 0.0001 in both groups). No statistically significant difference was evident between UC and CD patients. The mean serum TIMP-1 levels were significantly higher in patients with active IBD (450.5 ng/mL) as compared with patients with inactive disease (417.3 ng/mL, p = 0.03, Figure 2). Moreover, mean serum TIMP-1 levels proved to be significantly higher in IBD males (448.9 ng/mL), compared to IBD females (441.6 ng/mL, p = 0.01). Male gender was associated in all groups, HC included, with significantly higher mean serum TIMP-1 levels (399.8 ng/mL) compared to female gender (368.5 ng/mL, p = 0.04).

Mean (± SEM) serum TIMP-2 levels were 62.3 ± 2.3 ng/mL (median: 60.4, range: 33.5–112.0) in UC patients, 61.9 ± 2.4 ng/mL (median: 60.9, range: 0.6–106.0) in CD patients and 61.8 ± 4.5 ng/mL (median: 57.7, range: 2.0–187.7) in HC. No statistically significant difference was recorded between IBD patients and HC and this was also the case for patients with active and inactive disease (p > 0.05 in all cases).

Mean (± SEM) serum TIMP-4 levels were 1761.2 ± 67.7 pg/mL (median: 1658.1, range: 1089.3–2957.8) in UC patients, 1708.1 ± 73.4 pg/mL (median: 1655.1, range: 965.1–3605.9) in CD patients and 5573.4 ± 1246.3 pg/mL (median: 1364.4, range: 336.4–41.752.1) in HC, (Figure 3). UC and CD patients showed significantly lower serum TIMP-4 levels compared to HC (p = 0.008 and p = 0.02 respectively). The difference observed in TIMP-4 levels between UC and CD patients, as well as between patients with active and inactive disease proved to be non-significant. There was a trend toward lower mean serum TIMP-4 levels in males with IBD (1650.7 pg/mL), as compared with females (1797.2 pg/mL), but this difference was not statistically significant. Interestingly males, including HC had significantly lower mean serum TIMP-4 levels (2772.9 pg/mL), as compared with females (3299.0 pg/mL, p = 0.01).

Analyses of other subgroups did not reveal any statistically significant difference in the serum levels of TIMP-1, TIMP-2 and TIMP-4 when ever and never smokers, and patients with or without extraintestinal manifestations were compared (p > 0.05 in all cases). Treatment with 5-ASA alone or with additional corticosteroids, infliximab or immunosuppressive agents, did not appear to influence TIMPs' serum levels. Moreover, TIMPs' serum levels did not vary significantly among the subgroups in which patients where classified with regard to disease location and behavior of CD, or the extent of UC.

### TIMP-1/TIMP-4 ratio

The mean ± SEM ratio between TIMP-1 and TIMP-4 levels was 0.25 ± 0.01 ng/mL (median: 0.24, range: 0.03–0.47) in UC patients, 0.28 ± 0.02 ng/mL (median: 0.25, range: 0.005–0.66) in CD patients and 0.22 ± 0.03 ng/mL (median: 0.22, range: 0.003–1.00) in HC. CD patients (p = 0.036) but not UC (p = 0.174) had significantly higher TIMP1/TIMP-4 ratio compared to HC group. No significant difference was found between UC and CD patients (p = 0.247). Also, the aforementioned ratio was not different between patients with active IBD as compared with patients with inactive disease (p = 0.528).

### SAA and CRP levels

Patients with active IBD had statistically significant higher SAA levels, (8.66 ± 3.84, range: 0.2–84.4), as compared with the patients with inactive disease (6.25 ± 1.70, range: 0.10–58.80, p = 0.001). Similarly, patients with active IBD had statistically significant higher CRP levels, (2.27 ± 1.20, range: 0.75–31.15), compared to patients with inactive disease (1.10 ± 0.30, range: 0.00–19.20, p = 0.002).

### Correlation studies

The CRP levels were found to be significantly correlated with the levels of TIMP-1 (r = 0.247, p = 0.01) and TIMP-4 (r = 0.217, p = 0.03). Likewise, SAA levels were found to be significantly correlated with the levels of TIMP-1 (r = 0.264, p = 0.008) and TIMP-4 (r = 0.212, p = 0.03). A statistically significant correlation between TIMP-1 and TIMP-4 levels (r = 0.221, p = 0.02) was also observed. No correlations were evident between TIMP-1 or TIMP-4 serum levels and the age of patients or the duration of disease.

## Discussion

Our study provides new data on the tissue remodeling process in IBD with regard to serum TIMPs levels. We confirmed an increased production of TIMP-1 in IBD patients, especially in active disease and showed that this increase correlated with the production of the well-known inflammatory markers CRP and SAA. On the other hand, a decreased production of TIMP-4 was detected in these patients, which was independent of disease activity along with a strong correlation between TIMP-4 levels and the studied inflammatory markers. What seemed to be of particular interest was also the observation that males in comparison with females, tend to show an increased production of TIMP-1, accompanied by a decreased production of serum TIMP-4.

It has been shown that the uncontrolled and sustained inflammatory cascade, observed in IBD, gives rise to the production of MMPs and TIMPs which in turn can induce tissue degradation and lesion development [1,2]. We agree that the determination of serum MMP levels would add to the impact of this study. Unfortunately this was not included in the initial study design. The sera from these 150 patients are not available anymore, as they have been utilized for other determinations. On the other hand, it would be biased to obtain new sera because the inflammatory status (activity) of these patients has changed.

An increased production of TIMP-1 at the mucosa and plasma of IBD patients has been previously described [5-8]. It has also been demonstrated that in IBD, TIMP-1 is expressed by inflammatory cells, fibroblasts and vascular smooth muscle cells most prominently in actively inflamed areas in ulcer bases [7]. In an ex vivo study using tissue cultures of intestinal biopsies from IBD patients, TIMP-1 was detected in significant titers in inflamed mucosa of both CD and UC specimens, in contrast with uninflamed mucosa [5]. In the above-mentioned study, TIMP-1 showed strong correlation with proinflammatory cytokines IL-6, IL-1β and IL-10. In another study, an increased expression of TIMP-1 mRNA in inflamed and especially ulcerated colon mucosa of IBD patients was evident [6].

Increased levels of TIMP-1 have also been associated with the presence of fibrotic strictures in CD [11]. It is well known that, TIMPs are important markers of fibrosis. In addition, CD and especially its stenotic form are characterized by increased fibrosis. It was thus expected to find a more pronounced increase in TIMP-1 levels, in these subgroups. In our study, a trend towards higher TIMP-1 levels was observed in CD compared with UC patients which was not statistically significant. This could be attributed to the rather small number of patients which did not allow reliable subgroup analysis. In a recent study, TIMP-1 plasma levels in UC patients were found to correlate positively with scored endoscopic degree of mucosal injury, disease activity indices, clinical activity indices and CRP concentration, thus implying that TIMP-1 plasma concentration may be a possible biomarker of disease activity [8]. External stimuli such as growth factors, phorbol esters and cytokines (IL-6, IL-1 and IL-1b) are all well-known triggers of TIMP-1 expression in various cell types [4,12]. Erythropoietin (EPO) a hormone that also serves as a promoter of TIMP-1 secretion, is increased in IBD patients, as we have demonstrated in a previous study [13]. Thus, it is possible that EPO and TIMP-1 act in a collaborative manner and play a key role not only in the erythroid cell physiology but also in several pathophysiological events in IBD.

No statistically significant difference in TIMP-2 levels was recorded between IBD patients and HC, in our study. TIMP-2 mRNA levels measured by polymerase chain reaction (PCR) in biopsies from IBD patients, remained unchanged in inflamed and uninflamed mucosa, in the study of von Lampe et al [6]. Finally, no remarkable expression of TIMP-2 was observed in inflamed mucosa of IBD patients, in an immunohistochemical study by Arihiro et al [7]. However, it is not safe to draw any conclusions based solely on these data thus, this subject requires further studies.

One major finding presented in this study is that TIMP-4 serum levels are decreased in IBD patients, irrespective of disease activity. When interpreting this result many considerations need to be taken into account. TIMP-4, the newest member of TIMPs, is a tissue-specific regulator of EMC remodeling, promoting inhibition of the MMP-2 activation [3]. On the other hand, MMP-2 activity in IBD patients has been shown to be rather excessive and it could be held accountable for TIMP-4 consumption which in turn could result in lower TIMP-4 levels. The enhanced MMP-2 activity in IBD may be of significant importance, as it is a key player in proper wound healing, angiogenesis and re-epithelization, as well as in the regulation of epithelial barrier function of the intestine [1,2]. It has also been suggested that the increased TIMP-4 expression in normal heart tissue could explain why myocardial tumors are so rare [14]. On the other hand, there is evidence that low TIMP-4 levels result in an anti-apoptotic effect [15]. These reports, taken together with the data of the present study, may support the importance of low TIMP-4 levels in IBD-related tumorigenesis. Additionally, TIMP-4 has been shown to inhibit platelet aggregation, thus implying TIMP-4 involvement in the regulation of platelet aggregation and recruitment [16]. These platelet aggregation responses proved to be enhanced in IBD patients, even in inactive disease [17]. These findings suggest that, the decreased TIMP-4 serum levels observed in IBD patients, even those with inactive disease, have an aggravating influence on the platelet aggregation in IBD. To confirm these speculations, further studies are necessary. In any case, the imbalance of TIMP-1 and TIMP-4 serum levels observed herein, may reflect the paradoxical effects of these inhibitors, which may act as either promoters or suppressors in many important pathophysiological processes involved in inflammation and wound healing in IBD.

We have also showed that, gender has an influence on TIMP-1 and TIMP-4 serum levels. This gender-related difference may prove interesting but, how this is controlled is the subject of many differing lines of research. The involvement of TIMP-1 in steroidogenesis was first evidenced in Leyding cells and ovarian granulose cells and led to the conclusion that it could affect germ cell development, by regulating steroid concentrations in both males and females [18]. Moreover, it has been shown that TIMP-1 is not a prerequisite for overall germ cell development and that it could be substituted by one of the other TIMPs [19]. According to our results, TIMP-4 could be a candidate for such an action. In a recent study with a relatively small sample size, no significant difference in TIMP-1 plasma levels between females and males was recorded [20]. Given the fact however, that only a few TIMP-related studies with focus on gender have been published, future studies are required so that our findings could be clarified.

## Conclusion

In summary, our data may reflect the continuous matrix turnover occurring in IBD, a process strongly associated with an imbalance of TIMP-1 and TIMP-4 serum levels. The increase of TIMP-1 serum levels in active disease and their significant correlation with the production of known inflammatory markers such as CRP and SAA, indicate that TIMP-1 levels could be used not only for diagnostic purposes but also for the assessment of activity in IBD. These findings bring into question the potential role of TIMPs in the pathogenesis, activity and therapy of IBD, thus emphasizing the need for further studies on this subject. Perhaps, in the future, the modulation of MMP and TIMP activities as well as ECM turnover may become part of a more rational, mechanism-based therapeutic manipulation in IBD.

## Abbreviations

TIMPs: tissue inhibitors of metalloproteinases; TIMP-1: tissue inhibitor of metalloproteinase 1; TIMP-2: tissue inhibitor of metalloproteinase 2; TIMP-4: tissue inhibitor of metalloproteinase 4; IBD: inflammatory bowel disease; HC: healthy controls; UC: ulcerative colitis; CD: Crohn's disease; CRP: C reactive protein; SAA: serum amyloid A; ECM: extracellular matrix; MMPs: Metalloproteinases; IL-6: interleukin -6; IL-1β: interleukin-1β; IL-10: interleukin-10; EPO: erythropoietin.

## Competing interests

The authors declare that they have no competing interests.

## Authors' contributions

ANK designed the study, carried out data analysis, data interpretation, and drafted the manuscript. AIK participated in the practical performance, carried out sample analysis and helped to draft the manuscript. IPD, VDL and PIM participated in the practical performance and carried out data collection. ACM, participated in the practical performance and helped to draft the manuscript. AEG and SPP supervised research and performed critical review of the manuscript. All authors read and approved the manuscript.

## Pre-publication history

The pre-publication history for this paper can be accessed here:


